# Integrated care delivery and health care seeking by chronically-ill patients – a case-control study of rural Henan province, China

**DOI:** 10.1186/s12939-015-0221-8

**Published:** 2015-12-14

**Authors:** Leiyu Shi, Marty Makinen, De-Chih Lee, Ruth Kidane, Nathan Blanchet, Hailun Liang, Jinghua Li, Magnus Lindelow, Hong Wang, Shuangbao Xie, Jian Wu

**Affiliations:** Johns Hopkins Bloomberg School of Public Health, 624 N. Broadway, Baltimore, Maryland 21205 USA; Results for Development Institute, 1100 15th Street, NW, Washington, DC 20005 USA; Department of Information Management, Da-Yeh University, Changhua, 51591 Taiwan R.O.C.; Johns Hopkins Primary Care Policy Center, Baltimore, 624 N. Broadway, Baltimore, Maryland 21205 USA; Department of Social Medicine and Health Care Management, School of Public Health, Jilin University, 1163 Xinmin Street, Changchun City, Jilin China; The World Bank, 1225 Connecticut Avenue NW, Washington, DC 20433 USA; Bill & Melinda Gates Foundation, 500 Fifth Avenue North, Seattle, WA 98109 USA; Henan Provincial Health Bureau, Zhengzhou, China; School of Public Health, Zhengzhou University, Zhengzhou, China

**Keywords:** Primary care, Diabetes, Community health centers, Integrated care, Quality of care

## Abstract

**Objective:**

This study examined the impact of an Integrated Care Delivery intervention on health care seeking and outcomes for chronically-ill patients in Henan province, China.

**Methods:**

A case-control study was carried out in six health care organizations from two counties in Henan province, China. 371 patients aged 50 years or over with hypertension or diabetes who visited either community health centers or hospitals in the Intervention or Control Counties were systematically selected and surveyed on health care seeking behavior, quality of care, and pathway of care for their major chronic condition. Bivariate analyses were performed to compare quality and value of care indicators between patients from the Intervention and Control Counties. Multivariate analyses were used to confirm these associations after controlling for patients’ demographic and health characteristics.

**Results:**

Patients in both the Intervention and Control Counties chose their current health care providers primarily out of concern for quality of care (provider expertise and adequate medical equipment) and patient-centered care. Compared with the patients from the Control County, those from the Intervention County performed significantly better on almost all the quality and value of care indicators even after controlling for patients’ demographic and health characteristics. Significant associations between types of health care facilities and quality as well as value of care were also observed.

**Conclusion:**

The study showed that the Integrated Care Delivery Model was critical in guiding patients’ health care seeking behavior and associated with improved accessibility, continuity, coordination and comprehensiveness of care, as well as reducing health inequities and mitigating disparities for older patients with chronic conditions.

## Introduction

It is a rather common scene in China for patients to be in long lines at large hospitals waiting to make an appointment, while physicians in community health centers (CHCs) are waiting for patients to drop by. According to the fourth China National Health Services Survey in 2008, over half of all patients chose to go to large hospitals directly for medical services. Tertiary hospital bed utilization rate reached 104 % and second-tier hospital bed utilization reached 90 %, while bed utilization rate at primary hospital or CHCs was just about 60 % in 2012 [1].

In a 2008 World Health Report, primary care was promoted as a model for the provision of fair and efficient care [2]. Strong primary care systems were associated with reducing health inequities and mitigating disparities in health care utilization [2, 3]. In China, township health centers (THCs) and rural health stations (RHSs) are the main primary care institutions in rural areas [4]. In addition, the outpatient department of county hospitals in rural area also provide primary care services [5]. Until now a seamless reciprocal referral system between primary care institutions and hospitals has not been established in China [6]. No restriction is made in selecting medical institutions for primary care services [7], which resulted in growing demand for primary care in higher level institutions, as more and more rural residents bypassed RHSs or THCs to seek care in county hospitals or even tertiary hospitals in the urban area. According 2012 data, more than 36 % of the outpatient services happened in the hospitals [8]. However, the use of hospitals for primary care conditions has a number of adverse consequences: it reduces accessibility in terms of longer traveling and waiting time, weakens continuity due to limited patient-provider contact, and increases the costs for both patients and the health care system. It is especially burdensome for the chronically ill who tend to have greater need for seeking health care and whose conditions are more suitable for CHCs. Guiding patients concentrating at tertiary hospitals to community-based care is a central concern of Chinese health policymakers and a focus of the new round of Chinese health care reform.

**Fig. 1:**
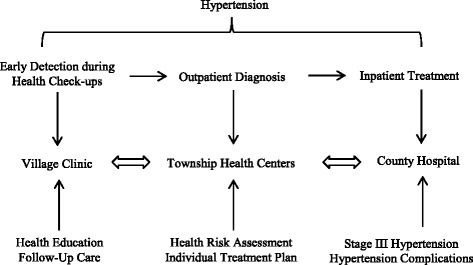
The integrated care delivery model for hypertension

For example, in Henan Province, the most populous and the largest agricultural province in China, with support from World Bank’s Rural Health Project [9], the government instituted an Integrated Care Delivery Model to promote appropriate health care utilization by improving access and coordination through the adoption of computerized clinical pathways, a shift from fee-for-service to case-based payment, performance-based payment for care providers, and Information technology (IT) -based monitoring on service quality of health care facilities. The overarching framework is a vertical referral system among different types of medical institutions. The system is designed to guide patients to appropriate medical institutions based on severity of diseases. Specifically, the hospitals would treat more complicated cases and township health centers and rural health stations (hereafter referred to as CHCs for simplicity) focus on primary care and chronic disease management. For patients, treatment would start from CHCs. The patients would then be referred either upwards to county hospitals or downwards to village clinics on the basis of severity of disease within a vertical system. As illustration, Fig. 1 depicts an Integrated Care Delivery Model for hypertensive patients across this primary care network. Another important intervention is a global payment system whereby CHCs as well as hospitals are paid based on patients’ clinical diagnoses and adherence to clinical pathways. The new model does not make it mandatory for patients to obtain care at a CHC first, but patients will pay significantly less copayment if seeking care from CHCs first. Patients referred by CHCs also get a significant discount in addition to receiving expedited treatment at county hospitals. Finally, the intervention includes an integrated information system, by which CHCs and hospitals share patients’ information. Table 1 compares the differences in the models of care between the Intervention and Control Counties.

**Table 1:** The main characteristics of models of primary care in the intervention and control counties

	Intervention county	Control county
Referral System	Vertical reciprocal referral system among different types of medical institutions	No apparent referral system arranged by the hospitals or CHCs
Care Management	Computerized clinical pathways	No clinical pathway
Payment System	Case-based payment	Fee-for-service
Incentive for Care Providers	Performance-based payment	No performance-based payment
Health IT System	Integrated information system whereby CHCs and hospitals share patients’ information	Internal information system at the hospital, but not connected to other facilities, such as the County Hospital or CHCs

The purpose of this study was to examine the impact of this Integrated Care Delivery intervention on access and care coordination for patients age 50 and over with chronic conditions and make suggestions for improving the efficiency, continuity, and effectiveness of chronic care at appropriate levels of the health care system. To the extent that patient health seeking behavior was altered and access and care coordination improved as a result of this intervention, the Henan experience could serve as a model for other rural Chinese provinces as well as other countries striving to improve their primary care delivery.

## Method

The case-control study method was used to carry out this study. Specifically, Xi County within Henan province was selected due to its intervention status. Huaibin County was selected as control due to its geographic proximity and population resemblance to Xi County. While the Integrated Care Delivery Model was implemented in Xi County, Huaibin County had no targeted intervention, other than changes implemented under the general health care reform^1^. The Intervention County is slightly larger in size with 336 villages (797,900 residents and 237,300 migrants) compared to 295 villages (581,000 residents and 150,000 migrants) at the Control County. However, per capita income is higher at the Control County than the Intervention County (RMB 19,640 vs. RMB 18,269). In terms of health status, while the two counties have comparable infant mortality (3.2–3.4 per 1000 live births) and diabetes (3 % each) rates, a higher proportion of the residents in the Intervention County have hypertension (13 % vs. 8 %) and infectious diseases (246 vs. 225 per 100,000) than in the Control County. In terms of health care resources, there are more health care facilities but fewer medical professionals and hospital beds in the Intervention County compared to the Control County2^2^.

We did not select standard impact evaluation as the study method, as it was not possible to randomly select a control group in advance, or identify a suitable comparison group through matching methods or use reflexive comparisons. However, statistical techniques were used to model the participation and outcome processes and to partially correct for selection bias in case-control study.

Within each county, we selected two settings as sites for data collection. These included county hospitals and CHCs. These sites were selected since they were the target health care facilities for the Integrated Care Delivery Model. The rationale was that if the intervention worked, patients at these sites would show greater improvement in care access and coordination in the Intervention County relative to the Control County. The selection of study sites was based on purposive sampling, with input from our local research partner, faculty from the Zhengzhou University. Specifically, one hospital and two CHCs were selected from each county.

### Study subjects

The study subjects were individuals age 50 or over with hypertension or diabetes who visited either CHCs or hospitals in the Intervention or Control Counties. These two conditions were selected because they were the most common chronic conditions affecting the elderly in China and most amenable to improved primary care.

### Data

Data for the study came from our field survey, and the technique in the collection of survey data was through face-to-face interview. The patients were selected in a systematic manner (i.e., every 5^th^ patient that met the selection criteria until the total quota was reached for that site). The sample size was calculated based on findings from a previous paper [10], and adjusted for site specific variations and refusal rate. Based on sample size calculation for survey respondents with 95 % confidence interval, 80 % power, and two locations, a minimum sample size of 80 patients was required for each type of facility (i.e., CHC and hospital), or a total of 320 patients for both counties (i.e., 80 from CHC and 80 from hospital per county). The actual sample size was 371, 51 more patients than minimally required (199 from Intervention County and 172 from Control County). Graduate students from the local Zhengzhou University School of Public Health conducted the survey, with on-site supervision from their faculty advisor and the project investigative team (jointly from Johns Hopkins University Primary Care Policy Center and Results for Development, a Washington DC-based non-profit analysis and research organization). Upon completion of the interview, each study subject was given a gift of daily necessity (e.g., toothpaste, soap, mug) valued at under US $5. The Human Subjects Research Committee of Zhengzhou University reviewed and approved the protocol of the study in compliance with the Declaration of Helsinki–Ethical Principles for Medical Research Involving Human Subjects.

### Measures

Although various conceptual models have been employed in studying health care seeking behavior, one of the most widely-used frameworks - the Behavioral Model of Health Services Use [11] - served as a foundation for our conceptual framework of health care seeking behavior. Specifically, we applied this framework to accomplish the objective of the study, i.e., to examine the impact of the Integrated Care Delivery intervention on health care seeking and quality for patients age 50 and over with chronic conditions.

According to this framework, health care use is influenced by both individual and system factors. Individual factors consist of predisposing, enabling, and need. Predisposing factors are factors that influence one’s inclination to use health care services, such as age, gender, occupation, ethnicity, education, and other demographic, social structure, and health belief factors. Enabling factors denote the availability of health care services and the ability of an individual to access services, such as health insurance, income, ability to travel, and distance to the nearest health care institutions. Need factors take health status into account by measuring existing disease, symptoms, general health status, disabilities, and other chronic health conditions. System factors include such characteristics of health care delivery as organizing, financing, and availability, and are reflective of the interventions associated with the Integrated Care Delivery Model. Based on these components of the conceptual framework, we developed independent and covariate measures for this study. These measures as well as their coding are shown in Table 2.

**Table 2:** Patient characteristics: intervention vs. control counties

	Intervention group (Xi County)				Control group (Huaibin County)			
	Total N	CHC 1	CHC 2	Hospital	Total N	CHC 1	CHC 2	Hospital
		N (%)	N (%)	N (%)		N (%)	N (%)	N (%)
Sample Size	199	50	57	92	172	46	46	80
Gender								
Male	92(46.23)	24(48)	26(45.61)	42(45.65)	70(40.70)	27(58.7)	30(65.22)	13(16.25)
Female	107(53.77)	26(52)	31(54.39)	50(54.35)	102(59.30)	19(41.3)	16(34.78)	67(83.75)
Age(Mean)	67.37(4.63)	61.5(1.22)	78.89(16.01)	63.41(1.10)	66.42(5.33)	61.13(1.35)	79.98(19.79)	61.68(1.28)
Marital Status								
Married	176(88.44)	44(88)	49(85.96)	83(90.22)	157(91.28)	44(95.65)	39(84.78)	74(92.5)
Divorced/widowed	23(11.56)	6(12)	8(14.04)	9(9.78)	15(8.72)	2(4.35)	7(15.22)	6(7.5)
Residence Status***								
Registered resident	188(94.47)	47(94)	51(89.47)	90(97.83)	105(61.05)	24(52.17)	40(86.96)	41(51.25)
Non-registered resident	11(5.53)	3(6)	6(10.53)	2(2.17)	67(38.95)	22(47.83)	6(13.04)	39(48.75)
Current Occupation***								
Farmer	173(86.93)	48(96)	53(92.98)	72(78.26)	110(63.95)	46(100)	39(84.78)	25(31.25)
Others	26(13.07)	2(4)	4(7.02)	20(21.74)	62(36.05)	0(0)	7(15.22)	55(68.75)
Highest Education**								
Primary school or below	146(73.37)	40(80)	45(78.95)	61(66.3)	103(59.88)	30(65.22)	32(69.57)	41(51.25)
Middle school or above	53(26.63)	10(20)	12(21.05)	31(33.7)	69(40.12)	16(34.78)	14(30.43)	39(48.75)
Per Capita Annual Income RMB (Mean)*	21981.9(1838.36)	19092.00	23592.98	22554.33	17291.98(1416.25)	8665.22	21573.91	19790.25
Type of Health Insurance***								
New rural cooperative medical insurance	177(88.94)	50(100)	57(100)	70(76.09)	116(67.44)	45(97.83)	44(95.65)	27(33.75)
Uninsured/self-pay/urban social insurance for workers/residents/public insurance	22(11.06)	0(0)	0(0)	22(23.91)	56(32.56)	1(2.17)	2(4.35)	53(66.25)
Current Health Status**								
Excellent/very good/good	22(11.06)	8(16)	7(12.28)	7(7.61)	36(20.93)	17(36.96)	8(17.39)	11(13.75)
Fair/poor	177(88.94)	42(84)	50(87.72)	85(92.39)	136(79.07)	29(63.04)	38(82.61)	69(86.25)
Chronic Conditions								
Hypertension or high blood pressure	152(76.38)	44(88)	45(78.95)	63(68.48)	138(80.23)	43(93.48)	38(82.61)	57(71.25)
Heart disease	60(30.15)	24(48)	10(17.54)	26(28.26)	50(29.24)	21(45.65)	8(17.39)	21(26.25)
Diabetes	83(41.71)	22(44)	21(36.84)	40(43.48)	77(45.29)	12(26.09)	13(28.26)	52(65)
Stroke^a^	3(1.51)	2(4)	0(0)	1(1.09)	14(8.19)	3(6.52)	1(2.17)	10(12.5)
Lung problems	14(7.04)	7(14)	1(1.75)	6(6.52)	11(6.40)	0(0)	6(13.04)	5(6.25)
Mental health problems^a^	5(2.51)	1(2)	1(1.75)	3(3.26)	2(1.16)	1(2.17)	1(2.17)	0(0)
Cancer^a^	2(1.01)	1(2)	0(0)	1(1.09)	1(0.58)	0(0)	1(2.17)	0(0)
Joint pain or arthritis	39(19.60)	18(36)	10(17.54)	11(11.96)	48(27.91)	7(15.22)	15(32.61)	26(32.5)
Other^a^	7(3.52)	1(2)	3(5.26)	3(3.26)	1(0.58)	0(0)	1(2.17)	0(0)
Number of Chronic Conditions	1.83(0.07)	2.4(0.15)	1.6(0.13)	1.67(0.09)	1.99(0.07)	1.89(0.12)	1.83(0.18)	2.14(0.08)

*p < 0.05 **p < 0.01, ***p < 0.001 based on t test continuous measures and Chi square test for categorical measures

^a^Chi square test was not available for variables with cell sample size less than 5

In addition, we conceptualize four dimensions of quality of primary care services and three aspects of values as represented in Starfield’s model of primary care [12]. The four quality dimensions are: accessibility, continuity, coordination, and comprehensiveness. The three aspects of value are satisfaction, cost, and health improvement. We included three dependent measures from each of the four quality dimensions, and two dependent measures from each of the three aspects of values. The study relied primarily on patients’ perceived quality and value of care rather than direct measures, as these measures would provide insights into both clinical and non-clinical outcomes that are important to patients and associated with patient-centeredness. These outcome measures and their coding are shown in Table 3.

**Table 3:** Quality & value of care: intervention vs. control counties

	Total Intervention Group	Intervention Group (Xi County)			Total Control Group	Control Group (Huaibin County)		
		CHC 1	CHC 2	Hospital		CHC 1	CHC 2	Hospital
	N(%) or Mean(SE)	N(%) or Mean(SE)	N(%) or Mean(SE)	N(%) or Mean(SE)	N(%) or Mean(SE)	N(%) or Mean(SE)	N(%) or Mean(SE)	N(%) or Mean(SE)
Sample Size Quality of Care	199	50	57	92	172	46	46	80
Access Get medical care in the evenings, on weekends, or holidays***								
Very easy	176(88.44)	42(84)	49(85.96)	85(92.39)	127(73.84)	40(86.96)	35(76.09)	52(65)
Somewhat easy/not sure	23(11.56)	8(16)	8(14.04)	7(7.61)	45(26.16)	6(13.04)	11(23.91)	28(35)
Satisfaction with Current Care Provider's Convenience (traveling time)***	4.73(0.05)	4.72(0.13)	4.72(0.11)	4.75(0.05)	4.37(0.06)	4.39(0.12)	4.52(0.07)	4.28(0.1)
Provider's Accessibility (access out-of-office hours by phone or text message)***	4.64(0.07)	4.12(0.22)	4.61(0.13)	4.93(0.03)	3.97(0.09)	4.07(0.13)	4.59(0.09)	3.56(0.15)
Continuity Healthcare professionals review with you all the medications***								
Yes	177(88.94)	45(90)	55(96.49)	77(83.7)	118(68.60)	41(89.13)	29(63.04)	48(60)
No/not sure/decline to answer	22(11.06)	5(10)	2(3.51)	15(16.3)	54(31.40)	5(10.87)	17(36.96)	32(40)
Health professionals always encourage you to ask questions***								
Always	190(95.48)	41(82)	57(100)	92(100)	105(61.05)	31(67.39)	28(60.87)	46(57.5)
Often/sometimes/rarely or never/NA/not sure/decline to answer	9(4.52)	9(18)	0(0)	0(0)	67(38.95)	15(32.61)	18(39.13)	34(42.5)
Healthcare professionals contact you to see how things are going***								
Yes	187(93.97)	42(84)	56(98.25)	89(96.74)	116(67.44)	39(84.78)	37(80.43)	40(50)
No/not sure/decline to answer	12(6.03)	8(16)	1(1.75)	3(3.26)	56(32.56)	7(15.22)	9(19.57)	40(50)
Coordination Coordinate your use of medications**								
Yes	183(91.96)	47(94)	55(96.49)	81(88.04)	141(81.98)	46(100)	35(76.09)	60(75)
No/Not Sure	16(8.04)	3(6)	2(3.51)	11(11.96)	31(18.02)	0(0)	11(23.91)	20(25)
Make referrals***								
Yes	146(73.37)	25(50)	43(75.44)	78(84.78)	86(50.00)	23(50)	24(52.17)	39(48.75)
No/Not Sure	53(26.63)	25(50)	14(24.56)	14(15.22)	86(50.00)	23(50)	22(47.83)	41(51.25)
Experienced coordination problems***								
No	141(70.85)	31(62)	40(70.18)	70(76.09)	81(47.09)	23(50)	22(47.83)	36(45)
Yes/Not sure/Decline to answer	58(29.15)	19(38)	17(29.82)	22(23.91)	91(52.91)	23(50)	24(52.17)	44(55)
Comprehensiveness Received secondary prevention services**								
Yes	166(83.42)	49(98)	49(85.96)	68(73.91)	131(76.16)	45(97.83)	25(54.35)	61(76.25)
No/Not Sure	33(16.58)	1(2)	8(14.04)	24(26.09)	41(23.84)	1(2.17)	21(45.65)	19(23.75)
Health professionals talk with you about things that can cause stress**								
Yes	174(87.44)	37(74)	51(89.47)	86(93.48)	131(76.16)	41(89.13)	39(84.78)	51(63.75)
No/have not seen a doctor in past 2 years/not sure/decline to answer	25(12.56)	13(26)	6(10.53)	6(6.52)	41(23.84)	5(10.87)	7(15.22)	29(36.25)
Health professionals talk with you about healthy diet or exercise^c^								
Yes	198(99.50)	50(100)	56(98.25)	92(100)	164(95.35)	44(95.65)	46(100)	74(92.5)
No/have not seen a doctor in past 2 years/not sure/decline to answer	1(0.50)	0(0)	1(1.75)	0(0)	8(4.65)	2(4.35)	0(0)	6(7.5)
Value of Care Satisfaction ^a^Total score of satisfaction with current care provider***	74.87(0.67)	71.64(1.84)	74.02(1.42)	77.15(0.44)	66.46(0.61)	64.39(1.22)	73.07(0.88)	63.85(0.75)
^b^Overall satisfaction with the care experience***	4.71(0.06)	4.44(0.17)	4.63(0.11)	4.9(0.03)	4.22(0.07)	4.43(0.11)	4.78(0.07)	3.78(0.1)
Cost Concern Satisfaction with out-of-pocket cost for chronic care***								
Payment very easily/easily afforded and affordable	181(90.95)	42(84)	53(92.98)	86(93.48)	107(62.21)	35(76.09)	32(69.57)	40(50)
Payment too high/way too high	18(9.05)	8(16)	4(7.02)	6(6.52)	65(37.79)	11(23.91)	14(30.43)	40(50)
Not receive the help you needed because of cost***								
No	162(81.41)	34(68)	46(80.7)	82(89.13)	100(58.12)	32(69.57)	21(45.65)	47(58.75)
Yes/not sure	37(18.59)	16(32)	11(19.3)	10(10.87)	72(41.86)	14(30.43)	25(54.35)	33(41.25)
Health Improvement Chronic condition relative to when it was first diagnosed***								
Significantly/somewhat improved	173(86.93)	39(78)	46(80.7)	88(95.65)	103(59.88)	32(69.57)	38(82.61)	33(41.25)
About the same/somewhat/significantly worsened	26(13.07)	11(22)	11(19.3)	4(4.35)	69(40.12)	14(30.43)	8(17.39)	47(58.75)
Experienced complications that required urgent attention**								
Yes	72(36.18)	14(28)	10(17.54)	48(52.17)	87(50.58)	17(36.96)	16(34.78)	54(67.5)
No/not sure	127(63.82)	36(72)	47(82.46)	44(47.83)	85(49.42)	29(63.04)	30(65.22)	26(32.5)

*p < 0.05 **p < 0.01, ***p < 0.001 based on t test for continuous measures and Chi square test for categorical measures

^a^This variable is the summary of the following items on patient satisfaction (each is coded as a 1-5 scale with 5 indicating most satisfied and 1 least satisfied): quality of care (equipment), quality of care (providers), patient-centered care, out-of-pocket cost, insurance plan requirement, choices of prescription drugs, traveling time, appointment time, waiting time, office opening hours, access out-of-office hours by phone or text message, coordination of needed services, comprehensiveness of services available or provided, referral from friends/relatives, and referral from a doctor

^b^This variable is worded as follows in the questionnaire: How satisfied are you with your overall health care experience? (coded as a 1-5 scale with 5 indicating most satisfied and 1 least satisfied)

^c^Chi square test was not available for variables with cell sample size less than 5

The survey was designed based on the framework to examine factors that influence patients’ health care seeking patterns and behavior and assess whether certain targeted interventions can modify patients’ health care seeking behavior and improve quality of care. Patients were surveyed on four sections: demographic information, health care seeking behavior, quality of care, and pathway of care for patient's major chronic condition. Demographic variables included age, gender, and socioeconomic and health status. The sections of health care seeking behavior and pathway of care for patient's major chronic condition included questions regarding patient’s health seeking patterns, their determinants, and factors that might influence or have influenced behavioral changes. In the section on quality of care, the questions were taken from the 2014 Commonwealth Fund International Health Policy Survey of Older Adults.

### Analysis

The overall aim of the analysis was to compare the quality and value of care by chronically-ill patients between Intervention and Control Counties. We conducted descriptive, bivariate, and multivariate analyses. First, we used Chi-square test to compare demographic and health profiles between subjects from the Intervention and Control Counties as well as across different health care settings. Next, we conducted bivariate analysis to compare quality and value of care indicators between subjects from the Intervention and Control counties. Last, we applied multivariate logistic regressions and multivariate linear regression to test the association between intervention and quality as well as value of chronic care after controlling for patients’ demographic and health characteristics. We used the survey commands to account for a clustered sample with six providers.

## Results

### Patient characteristics

Table 2 compares demographic and health profiles between subjects from the Intervention and Control Counties. Overall, a greater proportion of patients were females in both the Intervention and Control Counties (53.77 % and 59.30 % respectively). The average age of the participants was 67 and most were married. Most of the participants in Xi County were residents but a sizable from Huaibin County were migrants. Most were farmers and had primary school or below education. The per capita annual income was higher among hospital patients than CHC patients (RMB 19,790-22,554 yuan vs. RMB 8,665-23,593 yuan). Most of the participants in Xi County were covered under the new rural cooperative medical insurance (NRCMI) (88.94 %) but a sizable from Huaibin County were covered under other types of health insurance or uninsured (32.56 %). In terms of health status, hospital patients were more likely to consider themselves as of fair/poor health (92.93 % and 86.25 % in the Intervention and Control County, respectively) than CHC patients (63.04–87.72 %). Most patients had hypertension or diabetes.

### Reasons for choosing current health care providers

**Fig. 2:**
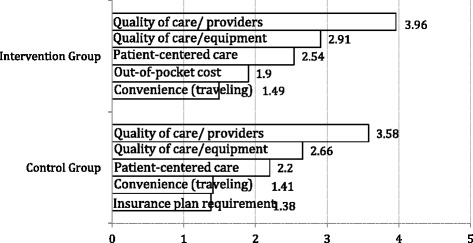
Top five reasons of choosing this facility

Figure 2 displays the top five reasons for choosing the current health care providers reported by patients from the Intervention and Control Counties. The figure depicts the scores on a scale from 1 to 5 with the top reason coded as 5, the next important as 4, and so on. Patients from the two counties had the comparable top three reasons: quality of care (provider expertise), quality of care (adequate medical equipment) and patient-centered care. Patients from the Intervention County reported out-of-pocket cost as their fourth reason for choosing the facility followed by convenience of traveling, while patients from the Control County reported convenience of traveling and insurance plan requirement as their fourth and fifth reasons, respectively. Further analysis showed that while CHC and hospital users shared their top reason: perceived quality of care (competence of providers and staff), they differed on other priorities. CHC users were more likely to care for patient-centered care (responsiveness/respect, privacy, time spent with the doctor, clear explanation of conditions) and convenience (traveling time). However, hospital users were more likely to care for perceived quality of care (equipment and facilities for diagnosis and treatment).

### Quality of care

#### Results from bivariate analyses

The first part of Table 3 shows 12 quality indicators that measure accessibility, continuity, coordination, and comprehensiveness of services. The Intervention County patients performed significantly better compared with the Control County, especially on the coordination and comprehensiveness domains. Specifically, patients from the Intervention County reported superior results with ratings above 90 % on the following indicators: health care professionals coordinate your use of medications (coordination domain), talk with you about healthy diet or exercise (comprehensiveness domain), always encourage you to ask questions (continuity domain), and contact you to see how things are going (continuity domain). Furthermore, most patients from the Intervention County did not experience coordination problems (70.85 %), while only 47.09 % patients from the Control County did not experience coordination problems. Likewise, health care providers in the Intervention County were more likely to make referral than the Control County (73.37 % vs. 50 %), and the difference in this measure was even greater between subjects from hospitals (84.78 % vs. 48.75 % in the Intervention and Control Counties, respectively). In terms of comprehensiveness of services, the Intervention County also had significantly higher rates than the Control County on the indicators of receiving secondary prevention services (84.42 % vs. 76.16 %), and health professionals talking with you about things that can cause stress (87.44 % vs. 76.16 %). Similarly, data from Table 2 indicates significantly better performance on the domains of access and continuity in the Intervention County than the Control County.

**Fig. 3:**
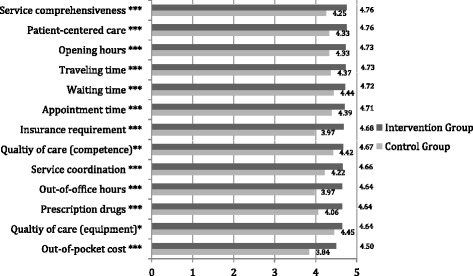
Patient satisfaction to current care provider (1–5 Likert Scale). *p < 0.05, **p < 0.01, ***p < 0.001

The relationship between intervention and patient satisfaction with the current care provider is displayed in Fig. 3. The figure visualizes the satisfaction scores of 13 indicators reported by patients from Intervention vs. Control Counties on a scale of 1 to 5 with 1 indicating least satisfied and 5 most satisfied. From this figure, it is apparent that patients from the Intervention County reported significantly higher score in all indicators (all the measures are at or above 4.50) than those in the Control County. Particularly, the most notable differences of scores between subjects from the Intervention and Control Counties were insurance requirement (4.68 vs. 3.97, *p* < 0.001), out-of-office hours (4.64 vs. 3.97, *p* < 0.001) and out-of-pocket money (4.50 vs. 3.84, *p* < 0.001).

#### Results from multivariate analyses

We fit multivariate logistic regression models to examine patient and institutional factors associated with quality of care for the chronic disease, controlling for patient demographic and health status characteristics (Table 4). Significant associations between the intervention and all the quality indicators were observed, with the exception of health professionals talking with you about diet or exercise. These results demonstrate that respondents in the Intervention County indicated that the system was more likely to perform well for them on the quality indicators that measure accessibility, continuity, coordination, and comprehensiveness of services. Specifically, the probability of patients from the Intervention County getting medical care in the evenings/weekends/holidays increased by 2.271 times (*p* < 0.05) compared with patients from the Control County. Patients from the Intervention County were more likely to report satisfaction with traveling time (OR: 5.694; 95 % CI: 3.241, 10.006) and access out-of-office hours by phone or text message (OR: 6.183; 95 % CI: 3.581, 10.678). In terms of continuity, health care professionals in the Intervention County were more likely to review all the medications (OR: 5.696; 95 % CI: 2.877, 11.278), encourage patients to ask questions (OR: 11. 936; 95 % CI: 5.348, 26.640), and contact patient to follow-up with care (OR: 6. 237; 95 % CI: 2.806, 13.866). Consistent with the results from the bivariate analyses, patients in the Intervention County reported significantly better quality of care in the domains of coordination and comprehensiveness in the multivariate analyses. The significant associations between type of health care facilities and quality of care were also observed. The results showed that health care providers in CHCs were more likely to review all the medications (OR: 2. 938; 95 % CI: 1.473, 5.860), coordinate with medication use (OR: 4.092; 95 % CI: 1.785, 9.382), and provide secondary prevention services (OR: 3.577; 95 % CI: 1.858, 6.887).

**Table 4:** Multivariate analysis: patient and institutional factors associated with quality of care for the chronic disease

	OR (95 % CI)					
	Access			Continuity		
	Get medical care in the evenings, on weekends, or holidays	Convenience (traveling time)	Accessibility (access out-of-office hours by phone or text message)	Healthcare professional review with you all the medications	Health professionals always encourage you to ask questions	Healthcare professionals contact you to see how things are going
Intervention group vs. Control group	2.271 * (1.164 4.432)	5.694 *** (3.241 10.006)	6.183 *** (3.581 10.678)	5.696 *** (2.877 11.278)	11.936 *** (5.348 26.640)	6.237 *** (2.806 13.866)
CHC vs. hospital	1.478 (0.734 2.976)	1.393 (0.786 2.468)	0.569 (0.311 1.041)	2.938 ** (1.473 5.860)	0.409 * (0.179 0.935)	0.923 (0.397 2.144)
Age (Mean)	0.999 (0.995 1.002)	1.002 (0.995 1.010)	0.999 (0.996 1.003)	1.022 (0.993 1.052)	0.999 (0.995 1.003)	1.003 (0.988 1.020)
Gender						
Female vs male	1.040 (0.541 2.001)	0.750 (0.434 1.296)	0.487 * (0.274 0.866)	1.140 (0.597 2.179)	0.587 (0.283 1.217)	1.436 (0.688 2.999)
Marital Status						
Married vs. divorced/widowed	1.816 (0.759 4.346)	1.158 (0.521 2.576)	1.023 (0.444 2.355)	0.112 * (0.014 0.876)	0.191 * (0.049 0.736)	1.126 (0.383 3.309)
Residence Status						
Registered resident vs. Non-registered resident	3.375 *** (1.676 6.794)	0.736 (0.387 1.401)	0.953 (0.498 1.822)	0.451 * (0.208 0.980)	1.427 (0.706 2.886)	0.590 (0.268 1.300)
Current Occupation						
Farmer vs. others	0.518 (0.218 1.230)	0.712 (0.349 1.451)	2.605 ** (1.278 5.308)	0.760 (0.344 1.676)	2.402 * (1.025 5.629)	12.327 *** (5.175 29.364)
Highest Education						
Middle school or above vs. primary school or below	0.837 (0.440 1.593)	1.696 (0.956 3.009)	0.822 (0.460 1.470)	0.950 (0.497 1.817)	1.222 (0.618 2.419)	1.187 (0.578 2.439)
Per capita annual income RMB	1.000 (1.000 1.000)	1.000 (1.000 1.000)	1.000 ** (1.000 1.000)	1.000 * (1.000 1.000)	1.000 * (1.000 1.000)	1.000 * (1.000 1.000)
Current Health Status						
Excellent/very good/good vs. fair/poor	2.405 (0.985 5.872)	1.156 (0.594 2.250)	0.956 (0.486 1.880)	2.382 (0.963 5.891)	2.949 * (1.205 7.218)	1.104 (0.438 2.780)
Number of Chronic Conditions	0.990 (0.718 1.366)	1.098 (0.842 1.431)	0.974 (0.749 1.265)	1.011 (0.743 1.376)	0.892 (0.648 1.226)	0.900 (0.633 1.280)
	OR (95 % CI)					
	Coordination			Comprehensiveness		
	Coordinate your use of medications	Make referrals	Didn’t experience coordination problems	Received Secondary Prevention Services	Health professionals talk with you about things that can cause stress	Health professionals talk with you about healthy diet or exercise
Intervention group vs. control group	3.644 ** (1.672 7.943)	2.352 *** (1.425 3.882)	2.722 *** (1.662 4.459)	2.797 ** (1.479 5.288)	2.663 ** (1.400 5.065)	2.009 (0.452 8.933)
CHC vs. hospital	4.092 *** (1.785 9.382)	0.575 * (0.335 0.988)	0.816 (0.488 1.364)	3.577 *** (1.858 6.887)	1.450 (0.743 2.830)	5.119 (0.960 27.290)
Age (Mean)	1.063 ** (1.024 1.103)	1.000 (0.997 1.003)	1.000 (0.997 1.003)	0.998 (0.995 1.001)	1.014 (0.985 1.045)	1.020 (0.946 1.099)
Gender						
Female vs. male	0.827 (0.366 1.870)	0.785 (0.467 1.319)	0.875 (0.532 1.440)	1.400 (0.761 2.578)	0.683 (0.355 1.314)	0.537 (0.095 3.043)
Marital Status						
Married vs. divorced/widowed	0.353 (0.044 2.846)	0.482 (0.209 1.109)	0.634 (0.291 1.381)	1.111 (0.421 2.931)	2.892 * (1.209 6.917)	<0.001 (<0.001 > 999.999)
Residence Status						
Registered resident vs. non-registered resident	0.127 *** (0.037 0.429)	1.703 (0.929 3.121)	1.059 (0.583 1.924)	0.655 (0.298 1.441)	0.467 (0.203 1.075)	<0.001 (<0.001 > 999.999)
Current Occupation						
Farmer vs. others	1.360 (0.517 3.577)	1.081 (0.558 2.094)	0.887 (0.470 1.675)	0.323 ** (0.138 0.756)	1.657 (0.740 3.710)	5.140 * (1.038 25.460)
Highest Education						
Middle school or above vs. primary school or below	1.486 (0.659 3.350)	1.380 (0.808 2.356)	0.867 (0.521 1.443)	1.738 (0.903 3.345)	2.301 * (1.118 4.739)	11.154 * (1.284 96.897)
Per Capita Annual Income RMB	1.000 ** (1.000 1.000)	1.000 (1.000 1.000)	1.000 (1.000 1.000)	1.000 * (1.000 1.000)	1.000 (1.000 1.000)	1.000 (1.000 1.000)
Current Health Status						
Excellent/very good/good vs. fair/poor	0.455 (0.180 1.155)	0.553 (0.300 1.018)	1.272 (0.686 2.360)	0.605 (0.292 1.253)	0.462 * (0.225 0.948)	>999.999 (<0.001 > 999.999)
Number of Chronic Conditions	0.721 (0.507 1.023)	0.893 (0.702 1.136)	0.820 (0.647 1.040)	0.850 (0.637 1.135)	1.089 (0.792 1.496)	1.072 (0.491 2.337)

*p < 0.05 **p < 0.01, ***p < 0.001

### Value of care

#### Results from bivariate analyses

Value of care was measured by satisfaction with care, concern over cost, and overall health improvement. The second part of Table 3 compares patients from the Intervention versus Control counties on these three aspects of value. First, in terms of satisfaction, respondents from the Intervention County reported significantly higher summary satisfaction score and overall satisfaction score than those from the Control County (74.87 vs. 66.46, 4.71 vs. 4.22, *p* < 0.001). Second, in terms of cost, compared with patients from the Control County, more patients from the Intervention County were satisfied with the out-of-pocket cost for their chronic care (90.95 % vs. 62.21 %, *p* < 0.001) and fewer patients did not receive medical care due to cost (18.59 % vs. 41.86 %, *p* < 0.001). Third, in terms of health improvement, compared to patients from the Control County, more patients in the Intervention County indicated improvement in their chronic condition relative to when it was first diagnosed (86.93 % vs. 59.88 %, *p* < 0.001) and fewer patients experienced complications that required urgent attention (36.18 % vs. 50.58 %, *p* < 0.01).

#### Results from multivariate analyses

Table 5 shows the results of multivariate analyses of patient and institutional factors associated with value of care for the chronic disease, controlling for patient demographic and health characteristics. We fit multivariate linear regression models to examine patient and institutional factors associated with total and overall scores of satisfaction with care. Similar to the results from the bivariate analyses, patients from the Intervention County reported significantly higher total as well as overall scores of satisfaction with care (*p* < 0.001), compared to those from the Control County. In particular, patients in the Intervention County scored an average of 7.6 more points on total satisfaction score than those from the Control County. They scored an average of 0.49 points higher on the overall satisfaction score than patients from the Control County. The rest of Table 5 displays multivariable logistic regression results examining factors associated with cost concern and health improvement with the chronic condition. Significant associations were observed between intervention status and concern over cost as well as overall health improvement. Specifically, the probability of patients from the Intervention County satisfied with out-of-pocket cost for chronic care increased by 5.769 times (*p* < 0.001) compared with patients from the Control County. Patients from the Intervention County were less likely to not receive care because of cost (OR: 2.901; 95 % CI: 1.678, 5.015), and more likely to indicate improvement in their chronic condition relative to when it was first diagnosed (OR: 6.773; 95 % CI: 3.651, 12.567). A significant association between type of health care facilities and value of care was also observed. The results showed that patients from CHCs were less likely to experience complications that required urgent attention than patients from hospitals (OR: 0.263; 95 % CI: 0.156, 0.446).

**Table 5:** Multivariate analysis: patient and institutional factors associated with value of care for the chronic disease

	Estimates (SE)		OR (95 % CI)			
	Satisfaction		Cost Concern		Health Improvement	
	Total Score of Satisfaction with Current Care Provider	Overall Satisfaction with the Care Experience	Satisfaction with Out-of-pocket Cost for Chronic Care	Receive the Help You Needed Despite the Cost	Chronic Condition Improved Relative to When it was First Diagnosed	Experienced Complications that Required Urgent Attention
Intervention group vs. control group	7.642(1.047)***	0.448(0.098)***	5.769 *** (2.849 11.680)	2.901 *** (1.678 5.015)	6.773 *** (3.651 12.567)	0.706 (0.420 1.187)
CHC vs. hospital	−0.641(1.062)	0.089(0.1)	1.915 (0.930 3.942)	0.692 (0.383 1.248)	1.185 (0.633 2.216)	0.263 *** (0.156 0.446)
Age (Mean)	−1.787(1.032)	0.001(0.001)	0.998 (0.994 1.003)	1.000 (0.996 1.004)	1.002 (0.995 1.009)	1.001 (0.998 1.005)
Gender						
Female vs male	0.007(0.007)	−0.241(0.097)*	0.762 (0.388 1.498)	0.786 (0.449 1.377)	0.609 (0.332 1.118)	0.960 (0.577 1.597)
Marital Status						
Married vs. divorced/widowed	−0.631(1.543)	−0.089(0.145)	2.245 (0.905 5.569)	1.071 (0.475 2.414)	0.691 (0.257 1.854)	1.021 (0.474 2.201)
Residence Status						
Registered resident vs. non-registered resident	−0.236(1.29)	−0.067(0.121)	3.309 *** (1.632 6.711)	2.085 * (1.116 3.894)	0.454 * (0.219 0.944)	0.417 ** (0.219 0.795)
Current Occupation						
Farmer vs. others	1.58(1.34)	0.132(0.126)	0.721 (0.306 1.696)	0.569 (0.272 1.191)	1.617 (0.776 3.368)	0.609 (0.314 1.182)
Highest Education						
Middle school or above vs. primary school or below	0.427(1.08)	−0.04(0.101)	2.293 * (1.129 4.658)	0.956 (0.545 1.677)	1.856 (0.981 3.509)	0.455 ** (0.260 0.798)
Per Capita Annual Income RMB	0.00004(0.00002)	0.000002(0.000002)	1.000 (1.000 1.000)	1.000 (1.000 1.000)	1.000 * (1.000 1.000)	1.000 (1.000 1.000)
Current Health Status						
Excellent/very good/good vs. fair/poor	−2.539(1.299)	−0.011(0.122)	1.225 (0.557 2.696)	1.323 (0.674 2.595)	2.997 * (1.279 7.020)	1.112 (0.578 2.139)
Number of chronic conditions	0.112(0.499)	0.021(0.047)	0.536 *** (0.388 0.741)	0.692 ** (0.534 0.896)	0.785 (0.592 1.040)	1.330 * (1.037 1.706)

*p < 0.05 **p < 0.01, ***p < 0.001

## Discussion

This study was one of the first to examine the impact of an Integrated Care Delivery intervention on quality and value of care for patients with chronic conditions in China. The study added evidence that implementation of Integrated Care Delivery Model could provide better primary care, and supported the appropriateness of the model in providing care to the chronically-ill patients. First, results from this study showed that patients in both the Intervention and Control Counties chose their current health care providers primarily out of concern for quality of care (both provider expertise and adequate medical equipment) and patient-centered care. Next, compared with patients from the Control County, those from the Intervention County reported that the system performed significantly better on almost all the quality and value of care indicators. Most of these indicators were still significantly better for the Intervention County patients even after controlling for patients’ demographic and health characteristics. Then, the significant associations between types of health care facilities and quality as well as value of care were also observed. Results showed that health care providers in CHCs were more likely to review all the medications, coordinate with medication use and provide secondary prevention services. Patients in CHCs were less likely to experience complications that required urgent attention than patients in the hospitals. Lastly, the results also showed that non-residents were associated with worse outcomes as measured by indicators of access to care during off-hours and cost concerns, but better outcomes as measured by review of medications, coordination of medications, improvement of chronic condition, and complications. The results regarding cost concerns indicated that non-resident populations seemed to face more financial barriers to care. One possible explanation for the worse access during “off hours” could be that most non-resident patients were rural migrant workers who were more likely to experience problems accessing information regarding how to get access to care in evenings, weekends, and holidays. However, the non-resident patients seemed to get better explanations of their medications than resident patients and benefit more from the care that they get than resident patients. Besides, non-residents were more likely in worse condition when they sought care, so they might benefit more when they got care.

These quantitative findings have been corroborated by a companion qualitative study (results available upon request) that showed patients could be referred back and forth within the vertical referral system across three settings of health facilities in the Intervention County, which largely improved quality and continuity of care. Referrals in the Control County were more sporadic and haphazard.

The study demonstrated that the implementation of Integrated Care Delivery Model was associated with improved accessibility, continuity, coordination and comprehensiveness of care, as well as reducing health inequities and mitigating disparities in health care utilization. In the Intervention County, reforms were introduced by adoption of reciprocal referral system, a shift from fee-for-service to global payment, performance-based payment for care providers, and integrated information system, by which CHCs and hospitals share patients’ information. Many previous studies showed the association between one aspect of improved quality of care and an individual intervention, for example, the seamless two-way referral system along with the incentive on lower copayment played a critical role in guiding patients concentrating at tertiary hospitals to community-based care, and effecting patients’ health care seeking behavior change [13–15]. Reforms carried out in the global payment and performance-based payment had provided incentives for health care providers to improve the quality and efficiency of care [14]. Moreover, the adoption of integrated information system facilitated continuity of care among multiple providers [16, 17]. Compared with above-mentioned findings, our study indicated that such interventions working together as our Integrated Care Delivery model led to more extensive improvement in health care quality and efficiency in the Intervention County to enhance continuity of care and coordinated services among different providers to address the needs of chronically-ill patients.

These study findings have provided policy and practice implications for China in its efforts to ensure equal access to affordable health services for the chronically-ill patients in rural area. The Integrated Care Delivery Model among county hospital, township health center, and village clinics served as a role model to provide continuity of care and coordinated services among different providers. For the Intervention County, further evaluation on the performance of the model is needed to examine the long-term impact and challenges in the process of reform. Policymakers would need to summarize replicable experience and support reforms in non-project areas, with the lessons learned to scale up the reforms all across China.

### Limitations

The current study had several limitations. First, the cross-sectional nature of the study made it difficult to make causal inferences from the analyses. The evidence on the impacts of the intervention is subject to possible biases from confounding factors, selection bias, and impact heterogeneity. Second, due to the pilot nature of the intervention, the study sites were selected only from one province, which limited the representativeness and generalizability of the study. Further research is needed to expand the investigation among multi-sites and to conduct prospective and experimental studies, such as using randomized clinical trials design. Third, the study examined patient perceived experiences rather than clinical or other more objective health outcomes. Future analyses could include clinical data to examine the health outcomes among patients with specific chronic illness. Last, the results of the analyses only showed that there were associations between the various measures of patient-reported improved care and the package of reforms. Due to the integrated nature of model conducted in the Intervention County. We could not separate out each component of the reforms.

## Conclusion

Despite these limitations, findings from this study are helpful in informing policy decisions and practice. This study is among the first to examine the association between Integrated Care Delivery intervention and quality as well as value of care in the rural area of China, providing an understanding of the impact of this new model on access and care coordination for older patients with chronic conditions, and making suggestions for improving chronic care at appropriate levels of the system. To face the challenges of a rapidly aging population and eruption of non-communicable disease epidemic, an adequately funded and well-organized primary care system can play a gatekeeping role and has the potential to provide a reasonable level of care to patients. Therefore, effective strategies include strengthening primary care to build a patient-centered health service delivery system and to provide more equitable, efficient, and high-quality health services.

## Abbreviations

CHCs: Community health centers
THCs: Township health centers
RHSs: Rural health stations
NRCMI: The new rural cooperative medical insurance

## Footnotes

1 The rapid aging of the population and the epidemic of non-communicable diseases call on new chronic care model which includes continuity of care and coordinated services among different providers to address the diverse needs of patients. Since the current health care reform which began in 2009, both Xi and Huaibin County within Henan Province have been actively engaged in reforming public health institutions and policies to improve quality, efficiency and cost control in chronic care delivery. With support from the World Bank-funded Rural Health Project between 2008 and 2014, Xi County was selected as a project area, and targeted intervention was implemented in Xi County to pilot the Integrated Care Delivery Model that aimed to integrate the services among county hospital, township health center and village clinics for patients with chronic conditions.

2 Source: Data form Xi and Huaibin Counties.
